# Clinical Course of Asymptomatic and Mildly Symptomatic Patients with Coronavirus Disease Admitted to Community Treatment Centers, South Korea

**DOI:** 10.3201/eid2610.201620

**Published:** 2020-10

**Authors:** Yong-Hoon Lee, Chae Moon Hong, Dae Hyun Kim, Taek Hoo Lee, Jaetae Lee

**Affiliations:** Kyungpook National University Hospital, Daegu, South Korea (Y.H. Lee, C.M. Hong, T.H. Lee, J. Lee);; Kyungpook National University, Daegu (Y.H. Lee, T.H. Lee, J. Lee);; Keimyung University Dongsan Medical Center, Daegu (D.H. Kim)

**Keywords:** respiratory infections, severe acute respiratory syndrome coronavirus 2, SARS-CoV-2, SARS, COVID-19, 2019 novel coronavirus disease, zoonoses, viruses, coronavirus, quarantine facility, community treatment center, mild, asymptomatic, RT-PCR, South Korea

## Abstract

We evaluated the clinical course of asymptomatic and mildly symptomatic patients with laboratory-confirmed coronavirus disease (COVID-19) admitted to community treatment centers (CTCs) for isolation in South Korea. Of 632 patients, 75 (11.9%) had symptoms at admission, 186 (29.4%) were asymptomatic at admission but developed symptoms during their stay, and 371 (58.7%) remained asymptomatic during their entire clinical course. Nineteen (3.0%) patients were transferred to hospitals, but 94.3% (573/613) of the remaining patients were discharged from CTCs upon virologic remission. The mean virologic remission period was 20.1 days (SD + 7.7 days). Nearly 20% of patients remained in the CTCs for 4 weeks after diagnosis. The virologic remission period was longer in symptomatic patients than in asymptomatic patients. In mildly symptomatic patients, the mean duration from symptom onset to virologic remission was 11.7 days (SD + 8.2 days). These data could help in planning for isolation centers and formulating self-isolation guidelines.

Coronavirus disease (COVID-19) is an infectious disease caused by a novel coronavirus, now called severe acute respiratory syndrome coronavirus 2 (SARS-CoV-2). COVID-19 has been spreading rapidly in many countries worldwide since the pandemic began in Wuhan, the capital of Hubei Province in China, in December 2019 (*1*,*2*). In South Korea, a confirmed case of COVID-19 was reported on January 20, 2020, and the number of confirmed cases has increased markedly since late February, especially in the Daegu and Gyeongsangbuk-do regions (*3*). Mass infection at a religious institution in Daegu City was the main cause of the surge in COVID-19 cases, which affected almost two thirds of the patients diagnosed in Daegu. To prevent further spread in the community, all members of this group were screened for SARS-CoV-2, regardless of whether they had symptoms. Real-time reverse transcription PCR (rRT-PCR) analysis of the nasopharyngeal swab samples from 10,459 persons showed 4,259 (40.7%) were positive for SARS-CoV-2 (*4*).

The exponential increase in COVID-19 cases in this area was so severe that local and government medical institutions were not able to handle the surge. Thus, many symptomatic patients, including some with advanced respiratory insufficiency, had to wait at home for hospitalization because no beds were available (*3*,*5*). In addition, hospital overload because of crowding with patients diagnosed with COVID-19 prevented adequate allocation of medical resources for patients with higher mortality risk because of age and presence of underlying conditions. To promote efficient allocation of advanced medical resources to severe COVID-19 patients, on March 2, 2020, South Korea implemented community treatment centers (CTCs), novel institutions to accommodate and monitor asymptomatic to mildly symptomatic case-patients who do not require hospital admission (*6*).

The clinical spectrum of COVID-19 could range from asymptomatic to severe pneumonia with respiratory failure and even death (*7*–*9*). Several studies have been published on the clinical characteristics or outcomes of COVID-19, but most analyzed data from hospitalized patients (*10*–*12*). To date, details of the natural course of COVID-19 in patients with no or mild symptoms in out-of-hospital settings has not been well documented. We describe the demographic and clinical characteristics of patients at 2 CTCs in Daegu, South Korea, and factors associated with treatment outcomes in such patients.

## Materials and Methods

### Study Design and Patient Classification

All patients included in the study tested positive for SARS-CoV-2 by rRT-PCR analysis of oral or nasal swab samples (*13*). After diagnosis, patients were isolated at home, and volunteer doctors interviewed patients through telephone calls >2 times a day. The volunteer doctors also performed risk assessment and patient classifications. Staff from the Department of Health and Welfare of Daegu Metropolitan City determined when and where patients would be admitted.

In South Korea, the COVID-19 risk assessment evolved gradually. During our study period, the Korea Centers for Disease Control and Prevention (KCDC) defined high-risk patients as persons >65 years of age, those with oxygen saturation <90% on room air, or those with chronic underlying diseases (*14*). KCDC guidelines also classified COVID-19 cases as asymptomatic, mild, severe, or very severe (*14*). 

We used KCDC guidelines to classify patients. Asymptomatic patients were defined as persons <50 years of age with no underlying conditions who were nonsmokers and had a body temperature of <37.5°C without taking antipyretic drugs. Mildly symptomatic patients were defined as persons <50 years of age with >1 underlying condition and a temperature of <38°C with antipyretic drugs. Severe patients were defined as persons who were alert but had dyspnea or temperature >38°C despite taking antipyretic drugs. Very severe patients were persons who had decreased alertness. 

After assessment by staff from the Department of Health and Welfare of Daegu Metropolitan City, severe, very severe, and high-risk patients were admitted to hospitals. Only asymptomatic or mildly symptomatic patients were admitted to CTCs. Because of the rapid surge of patients and hospital overload, however, some high-risk asymptomatic and mildly symptomatic patients were transferred to our CTCs.

Our study included patients treated at 2 CTCs during March 2–31, 2020. We collected data until April 12. We retrospectively collected data from electronic medical records (EMR) on patients’ age and sex; underlying conditions; clinical, laboratory, and radiographic findings; treatment; and outcomes. We defined respiratory symptoms as dyspnea, cough, sputum, rhinorrhea, or sore throat and gastrointestinal symptoms as diarrhea, dyspepsia, or constipation. The institutional review board of Kyungpook National University Hospital (KNUH) approved this study design and informed consent was waived (IRB no. 2020–04–038).

### CTCs

The 2 CTCs were existing facilities temporarily converted for patient isolation in Daegu. CTC1 was the Daegu National Education Training Institute, which had 160 rooms. CTC2 was a student dormitory of Kyungpook National University that had 480 rooms. Both CTCs were affiliated with KNUH in Daegu. During our study, the maximum number of patients per day was 150 at CTC1 and 383 at CTC2. Each patient had a separate room, except for families with young children, who stayed together. Patients were asked to remain inside their rooms during their entire admission to prevent spreading the infection.

CTC1 was open during March 2–April 30, 2020. CTC2 was open during March 8–28, 2020. Patients were transferred to CTC1 when CTC2 closed. KNUH dispatched doctors, nurses, medical technicians, and portable radiograph machines to each center and installed EMR and picture archiving and communication systems, from which authorized medical staff in KNUH could access data. Medical staff in CTCs and KNUH actively communicated with each other for patients’ care.

Patients were assessed by doctors and nurses through telephone 2 times every day. Body temperature and respiratory symptoms were routinely assessed by self-monitoring and reporting. If patients complained of symptoms, medical staff went to the patient’s room and examined them. Chest radiography and oxygen saturation measurement were performed at physicians’ discretion. Conservative treatment, such as antipyretics, was provided for mild symptoms, but patients who needed advanced medical care were transferred to the hospital.

### Laboratory Procedures

Physicians at the CTCs obtained nasopharyngeal and oropharyngeal swab specimens from patients for rRT-PCR testing. Specimens were sent to KNUH, and rRT-PCR analysis was performed for detecting SARS-CoV-2 by using Allpex 2019-nCoV Assay (Seegene Medical Foundation, https://www.seegenetech.com). A medical laboratory specialist interpreted results as negative, positive, or inconclusive.

Patients were assessed 5–7 days after admission to a CTC; if the rRT-PCR result was negative for SARS-CoV-2, another test was performed after 24 hours. If initial rRT-PCR result was positive, the next test was performed 3–5 days later. If the result was inconclusive, the next test was performed 2 days later. Patients were considered to be in virologic remission when 2 serial nasopharyngeal samples tested negative >24 h apart.

### Statistical Analysis

We noted continuous and categorical variables as mean + SD and no. (%). We defined the virologic remission period as the number of days from diagnosis to virologic remission. We performed a log-rank test to evaluate each factor related to hospitalization and virologic remission, such as age, sex, underlying conditions, and symptoms. We excluded patients transferred to the hospital from our analysis of virologic remission because their data were unavailable after transferred to hospital. We generated Kaplan-Meier curves for visualization of cumulative virologic remission rate of asymptomatic and mildly symptomatic patients. We analyzed data by using R version 3.6.3 software (https://www.r-project.org), and we considered p<0.05 statistically significant.

## Results

### Patient Characteristics

Among 640 patients treated at 2 CTCs, we excluded 8 from our analysis, 7 because we did not have enough data, and 1 because the patient was transferred from the hospital and discharged soon after symptom improvement. We analyzed data on 632 patients, 272 from CTC1 and 360 from CTC2. Among patients included, 430 (68.0%) were female and 202 (32.0%) male; the mean age was 40.6 years (SD + 17.3 years), and 112 (17.7%) patients had >1 underlying condition. After COVID-19 diagnosis, patients were self-isolated at home an average of 7.8 days (SD + 3.8 days) before admission to a CTC.

Among 632 patients, only 31 (4.9%) were symptomatic at diagnosis; 44 (7.0%) were asymptomatic at diagnosis but developed symptoms by the time they were admitted to the CTC. Among patients who were asymptomatic at the time of admission, 186 (29.4%) developed symptoms during CTC admission and 371 (58.7%) remained asymptomatic. During their illnesses, 187 patients (29.6%) had respiratory symptoms: 87 (13.8%) had cough, 78 (12.3%) had sputum, 45 (7.1%) had rhinorrhea, 45 (7.1%) had sore throat, and 10 (1.6%) had dyspnea. Fifty-four (8.5%) patients had gastrointestinal symptoms, such as abdominal pain or diarrhea; 30 (4.7%) patients had headache, 24 (3.8%) had fever, and 37 (5.9%) had other symptoms (Table 1).

**Table 1:** Characteristics of 632 patients with diagnosed coronavirus disease admitted to community treatment centers for isolation, South Korea*

Characteristics	Total	Hospitalized, n = 19	p value	Released or in remission, n = 578	p value
Sex					
M	202 (32.0)	6 (31.6)	0.9	187 (32.4)	0.1
F	430 (68.0)	13 (68.4)	Referent	391 (67.6)	Referent
Age, y					
Mean + SD	40.6 + 17.3		0.005†		0.5†
<20	44 (7.0)	2 (10.5)		38 (6.6)	
20–29	204 (32.3)	1 (5.3)		194 (33.6)	
30–39	74 (11.7)	0		70 (12.1)	
40–49	76 (12.0)	3 (15.8)		69 (11.9)	
50–59	118 (18.7)	5 (26.3)		105 (18.2)	
>60	116 (18.4)	8 (42.1)		102 (17.6)	
Underlying conditions					
None	520 (82.3)	10 (52.6)	Referent	482 (83.4)	Referent
>1 condition	112 (17.7)	9 (47.4)	<0.0001	96 (16.6)	0.7
Hypertension	55 (8.7)	4 (21.1)	0.05	47 (8.1)	0.9
Diabetes	12 (1.9)	0	0.5	10 (1.7)	0.6
Dyslipidemia	22 (3.5)	1 (5.3)	0.6	20 (3.5)	0.3
Respiratory disease	16 (2.5)	0	0.5	16 (2.8)	0.3
Heart disease	5 (0.8)	1 (5.3)	0.006	4 (0.7)	0.2
Other	43 (6.8)	7 (36.8)	<0.0001	35 (6.1)	0.8
Symptoms					
None	371 (58.7)	0	Referent	359 (62.1)	Referent
Any symptom	261 (41.3)	19 (100.0)	<0.0001	219 (37.9)	<0.0001
Respiratory	187 (29.6)	14 (73.7)	0.0001	155 (26.8)	<0.0001
Gastrointestinal	54 (8.5)	2 (10.5)	0.9	47 (8.1)	0.07
Headache	30 (4.7)	2 (10.5)	0.2	26 (4.5)	1.0
Fever	24 (3.8)	5 (26.3)	<0.0001	16 (2.8)	0.1
Other	37 (5.9)	5 (26.3)	0.0001	29 (5.0)	0.4

*Values are no. (%) patients except as indicated. p values calculated by using log-rank test. †Age >50 y.

Nineteen patients (3.0%) were transferred to the hospital (Table 2). Statistically significant correlations with transfer to hospital included age >50 years (p = 0.005), having >1 underlying condition (p<0.0001), and developing symptoms during the course of illness (p<0.0001). Among the 19 patients transferred to the hospital, 14 (73.7%) had respiratory symptoms, 2 (10.5%) had gastrointestinal symptoms, 2 (10.5%) reported headache, 5 (26.3%) had fever, and 5 (26.3%) had other symptoms or conditions, such as severe anxiety and pregnancy.

**Table 2:** Characteristics of 19 patients with coronavirus disease transferred from isolation in community treatment centers to hospitals, South Korea*

Age, y/sex	No. days from diagnosis to CTC admission	No. days in CTC	Underlying conditions	Reason for transfer
13/F	7	3	None	Severe anxiety
12/F	8	4	Allergic rhinitis	Severe anxiety
53/F	2	2	None	Severe cough, abnormality on chest radiograph, oxygen saturation 94%
42/F	5	18	None	Severe cough and sputum
64/F	3	3	None	Severe headache and chest discomfort
53/F	3	3	None	Severe cough, sputum, GGOs on chest radiograph
59/F	2	5	Claustrophobia	Severe cough, sputum, poor oral intake
70/F	8	22	Hypertension	Persistent fever (38.5°C) after medication
86/F	5	9	Hypertension	Old age, abnormality on chest radiograph
23/F	6	14	Pregnancy	Pregnancy
69/M	6	3	Dyslipidemia, arrhythmia, Parkinson’s disease	Dyspnea, underlying conditions	
67/F	3	3	Cerebral aneurysm, postoperative state	Dyspnea, abnormality on chest radiograph
51/M	4	2	Hypertension, hepatitis B, hepatocellular carcinoma, post-operative state	Oxygen saturation 82%
66/M	3	15	None	Severe anxiety
57/M	4	3	None	Oxygen saturation 88%, tachycardia
47/F	11	3	None	Dyspnea
49/F	4	7	Meniere’s disease, claustrophobia	Severe anxiety
60/M	11	23	None	Dyspnea
80/M	5	22	Hypertension	Old age, abnormality on chest radiograph
*CTC, community treatment center; GGOs, ground glass opacities.				

### Virologic Remission 

**Figure 1:**
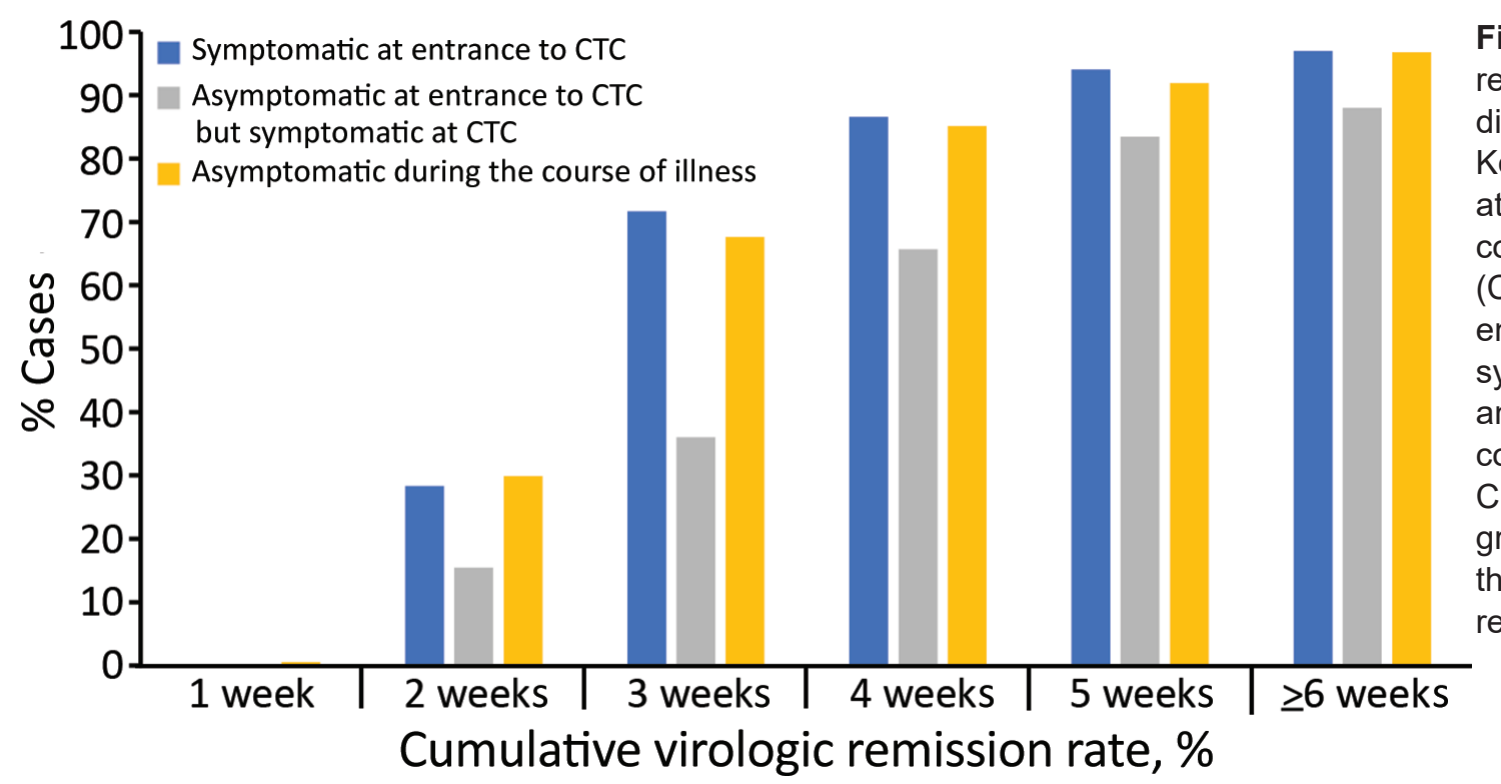
Cumulative virologic remission rate for coronavirus disease in patients in South Korea who were symptomatic at the time of entrance to a community treatment center (CTC), asymptomatic at the time of entrance to the CTC but developed symptoms during CTC admission, and asymptomatic during the course of illness after diagnosis. Cumulative remission rates of each group were calculated according to the time from diagnosis to virologic remission.

After excluding patients transferred to the hospital, 578/613 (94.3%) had virologic remission and were discharged from CTCs. A total of 2,522 rRT-PCR tests were performed (range 2–14/patient); 1,425 (56.6%) were negative, 733 (29.1%) were inconclusive, and 364 (14.5%) were positive. The virologic remission period was 20.1 days (SD + 7.7 days; range 7–45 days). Among 613 patients, 2 (0.3%) had virologic remission within 1 week, 157 (25.6%) within 2 weeks, 362 (59.1%) within 3 weeks, 489 (79.8%) within 4 weeks, and 550 (89.7%) within 5 weeks. Sixty-seven (10.9%) patients were symptomatic when they entered the CTC, 175 (28.5%) were asymptomatic at the time of admission but develop symptoms during isolation, and 371 (60.5%) remained asymptomatic (Figure 1). The virologic remission period was 19.0 days (SD + 7.4 days; range 7–43 days) for patients who were symptomatic at the time of entrance to the CTC, 23.1 days (SD + 7.7 days; range 8–45 days) for those who were asymptomatic at CTC admission but developed symptoms during isolation, and 19.1 days (SD + 7.5 days; range 7–45 days) for asymptomatic patients.

**Figure 2:**
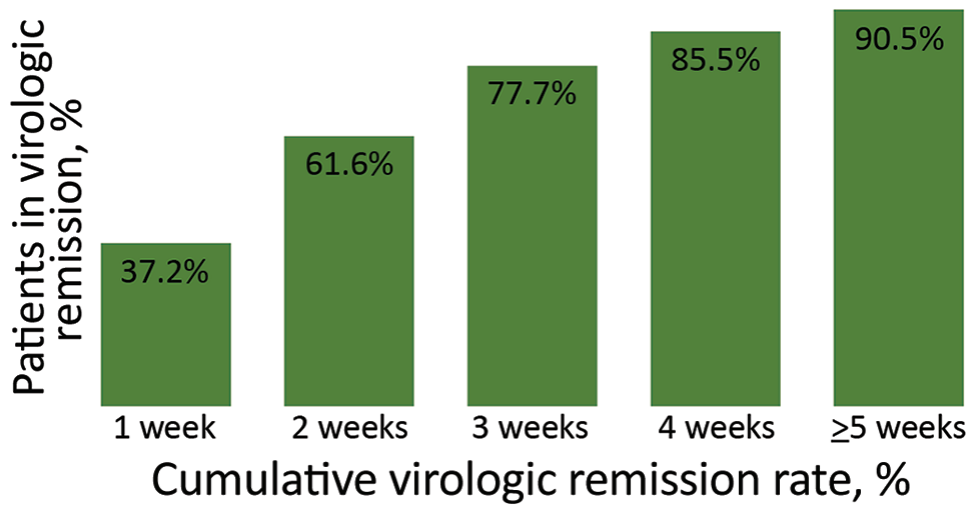
Cumulative virologic remission rate for coronavirus disease in mildly symptomatic patients in South Korea after symptom onset. Cumulative virologic remission rate of mildly symptomatic patients was calculated according to the time of the symptom onset to virologic remission.

Among 613 patients, 242 (39.5%) developed symptoms during their illness. The mean number of days from symptom onset to virologic remission for patients who developed symptoms was 11.7 days (SD + 8.2 days; range 2–41 days). Among 242 patients, 90 (37.2%) had virologic remission <1 week after symptom onset, 149 (61.6%) <2 weeks, 188 (77.7%) <3 weeks, 207 (85.5%) <4 weeks, and 219 (90.5%) during week 5 or longer (Figure 2).

**Figure 3:**
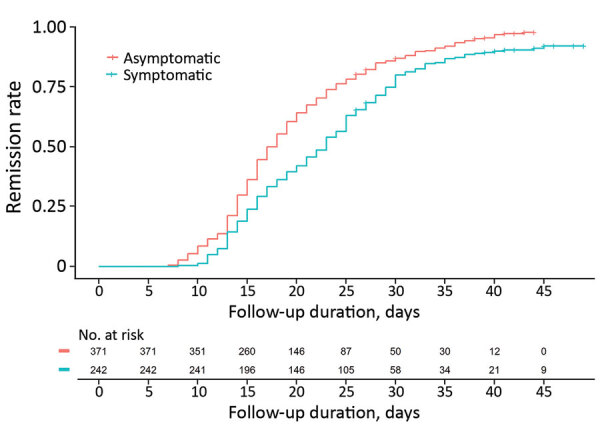
Virologic remission of coronavirus disease patients in South Korea according to symptoms. We noted a significant difference in virologic remission period between the asymptomatic and mildly symptomatic patients (p<0.0001).

Symptomatic patients had a longer remission period, 21.8 days (SD + 7.6 days), than asymptomatic patients 19.1 days (SD + 7.5 days; p<0.0001) (Figure 3). Respiratory symptoms had statistically significant correlation to the virologic remission period (p<0.0001). However, we noted no statistically significant differences in virologic remission period related to gastrointestinal symptoms (p = 0.07), headache (p = 0.1), fever (p = 0.1), and other symptoms (p = 0.4). We also saw no statistically significant differences in the remission period according to sex (p = 0.1), age (p = 0.5), or underlying conditions (p = 0.7).

## Discussion

We investigated the clinical characteristics and outcomes COVID-19 cases in asymptomatic and mildly symptomatic patients admitted for isolation and monitoring in 2 CTCs in South Korea. The mean duration from diagnosis to virologic remission was 20.1 days. For patients with mild symptoms, the virologic remission period was much longer than for asymptomatic patients (Figure 3).

The average age of the patients was ≈40 years; 68% were female and 32% were male. Demographic characteristics are linked to the population of religious institution in Daegu when CTCs were introduced, which seems to have had a demographic effect on our study population. Most (58.7%) patients remained asymptomatic during admission. Among patients with symptoms, cough and sputum were common, but fever, the most commonly observed symptom in studies involving hospitalized patients with COVID-19 (*9*,*10*), was less common in our patients, likely because of the CTC admission criteria. According to KCDC guidelines, patients with high fever or dyspnea were excluded from CTC admission because that might have required advanced medical treatment not available in CTCs (*14*).

A recent study that analyzed the viral dynamics of 76 hospitalized patients reported that severe COVID-19 cases tended to have viral loads ≈60 times higher than mild cases, with a longer viral shedding period (*15*). Although data on the relationship between clinical course and viral load in asymptomatic to mildly symptomatic patients with COVID-19 are lacking in our patients, the time from symptom onset to discharge was ≈12 days, which is ≈10 days shorter than the data from a study of hospitalized patients with COVID-19 (*8*). In terms of the natural course of COVID-19, this difference in recovery period suggests that the higher the disease severity and viral load, the longer it takes for virologic remission. Similarly, in our study, patients with symptomatic manifestation had a greater delay in the virologic remission period compared with asymptomatic patients. In particular, patients who were asymptomatic at admission to a CTC but symptomatic during follow up tended to have a longer virologic remission period, suggesting this patient subgroup might have peaked in disease severity or viral load during CTC admission. Therefore, even patients who are asymptomatic at the time of CTC admission should be followed closely to determine if they experience symptoms and then carefully managed during admission.

We had limited resources to perform rRT-PCR and could not perform daily testing for all obtained oral and nasal swabs samples. However, we did perform 2,522 rRT-PCR tests for early discharge from the center. In addition to the detection of SARS-CoV-2 in samples from the respiratory tract, viral RNA detection in stool samples and the possibility of active replication in the gastrointestinal tract have been suggested (*16*), but these tests were not clinically feasible in our facilities. A recent study reported virologic analysis of 9 cases of COVID-19 after the first week of symptoms but found no live virus isolates despite ongoing high concentrations of viral RNA (*16*). Therefore, further studies are needed to verify the relationship between viral RNA detected by rRT-PCR and infectivity of SARS-CoV-2.

Patient isolation is essential for preventing the spread of COVID-19. For practical operation of CTCs, we need to estimate the stay of the patients. Our results show that 59.1% of patients showed virologic remission at 3 weeks after diagnosis. In addition, ≈20% of the patients remained in CTC for >28 days after diagnosis. Combined with the fact that symptomatic patients took longer to discharge than asymptomatic cases, these data might be helpful for planning the establishment of isolation centers and formulation of self-isolation guidelines.

Nineteen (3.0%) patients at CTCs were transferred to hospitals, similar to recently reported data from another CTC in South Korea (*6*). The patients who were transferred were generally >50 years of age with underlying conditions, and the main reasons were worsening respiratory symptoms or abnormalities on chest radiographs. Although a CTC is not strictly a medical institution, we deployed a mobile radiology facility to our centers to help screen patients with pneumonia. In addition to symptoms caused by SARS-CoV-2 infection, 4 patients were transferred because of severe anxiety; 2 were adolescents and reported severe anxiety during isolation and separation from families. A previous report also had a case of hospital transfer due to serious psychiatric problems, including suicidal ideation (*6*). Therefore, successful CTC operation requires an established system capable of early detection of psychological symptoms and consultation with a psychiatrist.

Recent studies reported that age >65 years and presence of underlying conditions are the factors most related to outcomes for patients with COVID-19 (*11*,*12*). However, the time from diagnosis to remission in our study did not differ by age group. In asymptomatic or mildly symptomatic patients, as in our study population, the effect of age or underlying disease in terms of viral clearance might be minimal and further research is needed. However, in terms of hospitalization after clinical deterioration, older persons and those with underlying conditions tended to be more vulnerable and should be carefully monitored.

Our study has several limitations. First, CTCs did not have the capacity for the meticulous medical record keeping found in a hospital, and laboratory tests other than rRT-PCR for SARS-CoV-2 were not available. Second, we do not have data on reinfection or reactivation after discharge. Last, because timing of SARS-CoV-2 infection before diagnostic testing is unclear, the actual time interval from the date of the infection to virologic remission could be longer than we report in our study.

In conclusion, our results demonstrate the natural course of COVID-19 in a large population of asymptomatic and mildly symptomatic patients. These data might be helpful for planning isolation centers and formulating self-isolation guidelines for the public.
